# A Novel Extracellular Gut Symbiont in the Marine Worm *Priapulus caudatus* (Priapulida) Reveals an Alphaproteobacterial Symbiont Clade of the Ecdysozoa

**DOI:** 10.3389/fmicb.2016.00539

**Published:** 2016-04-26

**Authors:** Paul Kroer, Kasper U. Kjeldsen, Jens R. Nyengaard, Andreas Schramm, Peter Funch

**Affiliations:** ^1^Section for Genetics, Ecology, and Evolution, Department of Bioscience, Aarhus UniversityAarhus, Denmark; ^2^Section for Microbiology and Center for Geomicrobiology, Department of Bioscience, Aarhus UniversityAarhus, Denmark; ^3^Stereology and Electron Microscopy Laboratory, Department of Clinical Medicine, Centre for Stochastic Geometry and Advanced Bioimaging, Aarhus UniversityAarhus, Denmark

**Keywords:** Rickettsiales, invertebrate-bacteria symbiosis, Ecdysozoa, microvilli, *Priapulus caudatus*, gut symbiont

## Abstract

*Priapulus caudatus* (phylum Priapulida) is a benthic marine predatory worm with a cosmopolitan distribution. In its digestive tract we detected symbiotic bacteria that were consistently present in specimens collected over 8 years from three sites at the Swedish west coast. Based on their 16S rRNA gene sequence, these symbionts comprise a novel genus of the order Rickettsiales (Alphaproteobacteria). Electron microscopy and fluorescence *in situ* hybridization (FISH) identified them as extracellular, elongate bacteria closely associated with the microvilli, for which we propose the name “*Candidatus* Tenuibacter priapulorum”. Within Rickettsiales, they form a phylogenetically well-defined, family-level clade with uncultured symbionts of marine, terrestrial, and freshwater arthropods. *Cand*. Tenuibacter priapulorum expands the host range of this candidate family from Arthropoda to the entire Ecdysozoa, which may indicate an evolutionary adaptation of this bacterial group to the microvilli-lined guts of the Ecdysozoa.

## Introduction

*Priapulus caudatus* (Lamarck 1816) is a marine worm inhabiting soft bottom sediments. It belongs to the Priapulida, a small phylum with 19 known extant species (Schmidt-Rhaesa, 2012). The priapulids have a long fossil history with so-called stem-priapulids dating back to the mid-Cambrian (e.g., Huang et al., 2004), and some species, including *P. caudatus*, show little apparent morphological change since the Cambrian. The phylum belongs to the Scalidophora along with Kinorhyncha and Loricifera nested inside the major protostome taxon Ecdysozoa, the molting invertebrates (Aguinaldo et al., 1997). The members of Ecdysozoa are most notably characterized by the regular shedding of a cuticle (ecdysis) and comprise, among smaller phyla, the well-known arthropods and nematodes (for an overview, see Figure S1). The taxon Ecdysozoa is important for understanding the basal phylogeny of animals and has become well-established in metazoan taxonomy (e.g., Edgecombe et al., 2011).

*P. caudatus* can be found all over the northern hemisphere in low abundance and is an opportunistic predator and scavenger, preying on other invertebrates such as annelids and crustaceans (Vannier, 2012). Although it is the earliest described and most studied priapulid (Schmidt-Rhaesa, 2012), and despite the important role of symbiotic bacteria in a wide range of invertebrate hosts (reviewed in e.g., Dubilier et al., 2008; Nyholm and Graf, 2012), there is so far only a single report addressing a putative microbial symbiosis in *P. caudatus*: by transmission electron microscopy (TEM), McLean (1985) observed elongate bacterial cells associated with the microvilli in the intestinal lumen of two specimens of *P. caudatus* from Orcas Island, Northwest Pacific, USA. Bacteria were also observed within vacuoles of host intestinal cells but it could not be determined if these were different bacteria or whether they were undergoing digestion. While gut symbionts in certain arthropods, especially insects such as aphids and termites have been well investigated (Engel and Moran, 2013; Hansen and Moran, 2014), almost nothing is known about identity and function of gut symbionts in scalidophorans, including *P. caudatus*. The goal of the present study was to localize and identify these putative intestinal symbionts by 16S rRNA gene cloning and sequencing, TEM, and fluorescence *in situ* hybridization (FISH).

## Materials and methods

### Living material, sampling, and fixation

**Figure 1:**
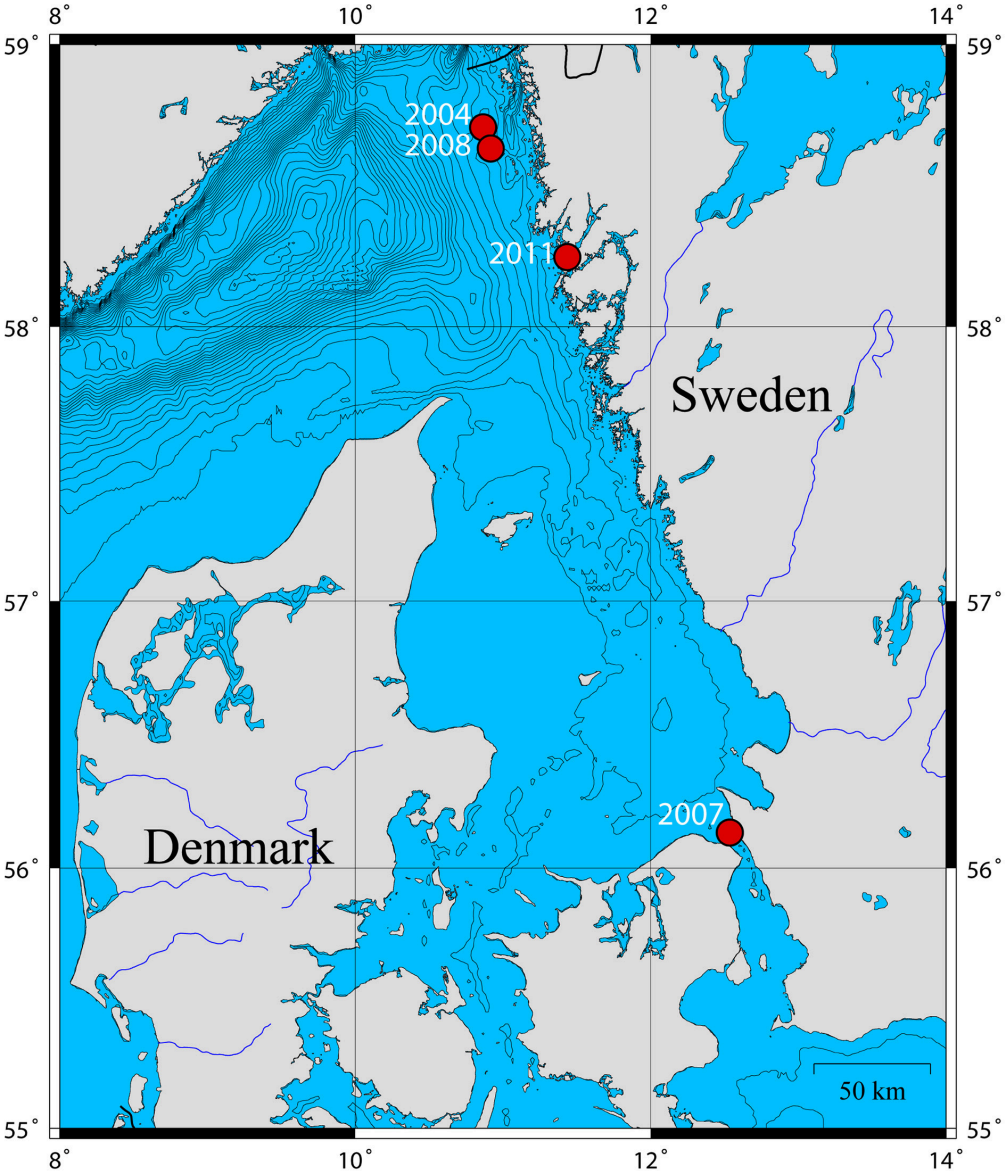
**Map of the ***P. caudatus*** sampling sites on the Swedish west coast and their respective sampling years**. Isopleths are spaced by depths of 25 m.

Two adult *P. caudatus* specimens were collected in 2011 from soft sediments at 35–60 m water depth from Gullmarsfjorden (Fiskebäckskil), Sweden (Figure 1) with a ring dredge. They were dissected in filtered sea water; the gut was cut open longitudinally and rinsed thoroughly to remove gut content. The gut of one specimen was fixed for TEM in 2% glutaraldehyde buffered with 0.2 M sodium cacodylate and 0.275 M sucrose for 3 h at 5°C (pH at 7.4), washed in 0.27 M sodium cacodylate with 0.37 M sucrose, post-fixed in 1% osmium tetroxide for 1 h and stored in ethanol at −18°C. The other gut was split longitudinally into two subsamples; one was fixed for FISH in 4% (w/v) paraformaldehyde in phosphate buffered saline (PBS, 130 mM NaCl, 10 mM Na-phosphate buffer, pH 7.4) at 4°C for 1 h, washed in PBS, and stored in ethanol at −20°C, the other was preserved for DNA extraction in TE buffer (10 mM Tris, 1 mM EDTA, pH 8.0). Mid-gut tissue samples from three *P. caudatus* specimens preserved in ethanol were obtained from Gothenburg Natural History Museum for additional DNA extractions. These specimens had been sampled from three different locations on the Swedish west coast in the years 2004, 2007, and 2008 (Figure 1).

### DNA extraction

DNA was extracted from the fresh samples using the DNeasy Blood and Tissue kit (Qiagen) according to the manufacturer's protocol. Ethanol-fixed samples were extracted in duplicate using the same kit with the following modifications: residual ethanol was removed by evaporation at room temperature prior to extraction; DNA yield was maximized by (i) incubating the eluent buffer (50 μl) in the DNA-binding filter column for 10 min, (ii) repeating the elution twice, and (iii) combining all eluent fractions from the duplicate extractions and concentrating the DNA extract to a final volume of 100 μl in a vacuum centrifuge (miVac DNA Concentrator, GeneVac ltd.).

### PCR, cloning, and sequencing

Near full-length 16S rRNA genes were amplified in 27 PCR cycles with *Taq* Master Mix RED (Ampliqon A/S) and the general bacterial primer 8F with ambiguities, 616V according to Juretschko et al. (1998) (5′-AGA GTT TGA TYM TGG CTC AG-3′) and universal primer Uni1492R (5′-GGY TAC CTT GTT ACG ACT T-3′; Loy et al., 2002) at an annealing temperature of 52°C. PCR products were cloned into *E. coli* JM109 using the pGEM-T vector system (Promega), and 168 clones were selected (96 from the fresh 2011 sample and 24 clones from each of the ethanol fixed samples) for partial Sanger sequencing (GATC Biotech). A few clones representing the phylotype of the putative symbiont were selected from each clone library for almost full-length sequencing. The 16S rRNA gene sequences of the symbiont were deposited in GenBank under the accession numbers KP138714, KP138715, KP138716 (full-lengths), and KU195266 (900 bp).

### Phylogenetic analysis

Sequences were trimmed to remove primer and vector bases, aligned with the SINA web aligner (Pruesse et al., 2012) and added to the alignment of the SILVA SSURef v115 ARB database (Quast et al., 2013) along with their most closely related sequences as identified by the basic local alignment search tool (BLAST; Johnson et al., 2008); the alignments were corrected manually in ARB (Ludwig et al., 2004). Representative sequences were further analyzed using Bayesian Inference (BI) and Maximum Likelihood (ML) methods. BI analysis was performed in MrBayes (Huelsenbeck and Ronquist, 2001), applying a general time-reversible (GTR) model of substitution with a proportion of invariable sites and gamma distributed among-site rate variation (GTR+I+Γ). The number of generations was set to 3 million with a sampling and printing frequency of 200. All other settings remained at default, i.e., two runs were carried out in parallel. Runs converged at a standard deviation of 0.0034, and the resultant full tree is shown in Figure S2.

**Figure 2:**
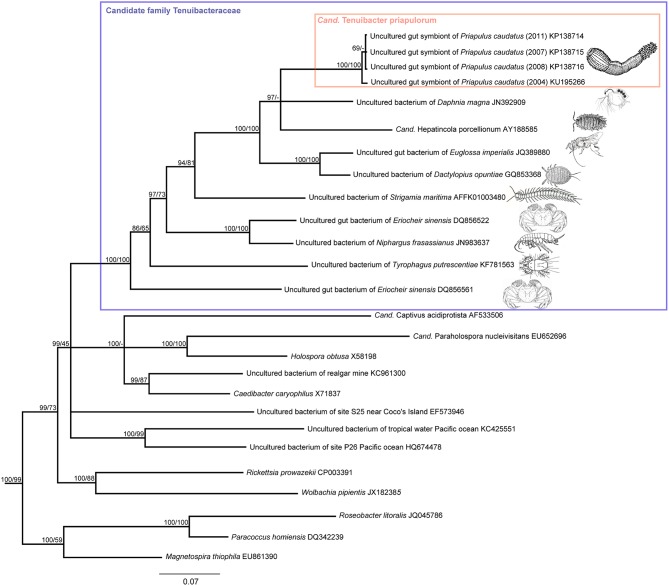
**Phylogenetic affiliation of the ***P. caudatus*** symbionts within the proposed candidate family Tenuibacteraceae and relative to other Alphaproteobacteria based on 16S rRNA gene sequence data**. Consensus tree of maximum likelihood (ML) and Bayesian inference (BI) analyses; inconsistent branchings are shown as multifurcations. Node values indicate posterior probabilities (left) and ML bootstrap values (right). Branch lengths were based on BI; scale bar, 7% estimated sequence divergence.

ML analysis was performed with RaxML v. 7.0.3 (Stamatakis, 2006) implemented in the ARB software. A GTR+I+Γ model was chosen with rapid bootstrap analysis of 5000 runs. The resultant full phylogenetic tree is shown in Figure S3. A simplified consensus tree was constructed based on the BI tree and incongruences with the ML tree are shown as multifurcations (Figure 2). Tree visualizations were done using the FigTree software (v1.4).

### Light microscopy and TEM

The glutaraldehyde fixed sample was dehydrated using graded ethanol series and acetone and embedded in an epoxy resin (kit 812 (Epon) from TAAB Laboratories Equipment ltd.) using acetone as an intermedium, and sectioned with an ultramicrotome. Semi-thin sections were stained with toluidine blue and mounted on microslides for light microscopy on an Olympus BX60 with an attached Olympus XC30 camera. Micrographs were composed using the multi-image alignment tool of the CellSens 1.7 software (Olympus Soft Imaging Solutions GmbH).

Ultrathin sections were collected on Formwar-coated grids with 1 × 2 mm slots, stained with Ultrostain-1 (stabilized solution with 0.5% uranyl acetate; Leica microsystems) for 10 min, rinsed in distilled water, stained for 5 min with lead nitrate/sodium citrate (pH 12; Ampliqon A/S), and examined on a Phillips Morgani 268 microscope using a high voltage of 80 kV; digital micrographs were obtained with an attached MegaView III SIS camera.

### FISH

Using the probe design feature of the ARB software, an oligonucleotide probe, PricSym652 (5′-TATCCCCTT CTGTTCTCT-3′, spanning *E. coli* 16S rRNA gene positions 652–669), was designed to specifically target the 16S rRNA of the putative *P. caudatus* symbiont. Probe specificity and hybridization characteristics were evaluated *in silico* using the “probecheck” tool (Loy et al., 2008) and the “mathFISH” software (Yilmaz et al., 2011). The probe had two central mismatches to the closest non-target sequence in the database, a Firmicutes-affiliated clone sequence. A melting formamide concentration, [FA]_m_, of 46.2% at 46°C in standard FISH buffer was predicted by mathFISH, and FISH with a formamide series (40–60% with increments of 5%) confirmed signal drop-off >45% formamide; therefore 45% formamide was chosen for specific hybridization.

The paraformaldehyde-fixed sample was washed in PBS and divided into several subsamples, which were embedded overnight in Tissue Freezing Medium® (Jung) and sectioned at −14°C on a cryomicrotome (Leica CM 1850). The 10 μm thick sections were collected on SuperFrost® slides, washed in Milli-Q water (Millipore) to remove the embedding, and dehydrated in a graded ethanol series. A ring was drawn on the slides around the tissue section with a hydrophobic pen (Pap-Pen, Kisker) to create a hybridization well. DOPE-FISH was performed as previously described (Stoecker et al., 2009), with hybridization times of at least 2 h. Probes EUB338, EUB-II, EUB-III (Daims et al., 1999), and NON (Manz et al., 1992) were used as positive and negative controls, and probe ALF1b (Manz et al., 1992) to ensure specific detection of the alfaproteobacterial symbionts; all probes were synthesized and double-labeled with CY3 or CY5 by Biomers (biomers.net). Hybridized samples were mounted in Citifluor/Vectashield (4:1) containing 1 μg ml^−1^ of DAPI and imaged on an Axiovert 200 M microscope (Carl Zeiss) with attached AxioCam MRm camera and Apotome module, using AxioVision 4.8 software.

## Results and discussion

### A specific symbiont in the midgut of *Priapulus caudatus*

Each of the clone libraries derived from four *P. caudatus* specimens collected in 4 different years at three distinct sites at the Swedish west coast were strongly dominated by a single phylotype, with all 96 clones, or 24, 22, and 21 of the 24 clones analyzed, showing ≥99.3% 16S rRNA gene sequence identity. The closest described relative (88% sequence identity) of the *P. caudatus*-specific phylotype was “*Candidatus* Hepatincola porcellionum,” an uncultured extracellular symbiont in the midgut gland (hepatopancreas) of the rough woodlouse *Porcellio scaber* (Wang et al., 2004), and a member of the alphaproteobacterial order Rickettsiales (Figure 2). The most similar sequences from environmental (non-host-associated) sources were from marine waters but were only < 84% identical to the *P. caudatus* phylotype (Figure 2). Only three additional phylotypes were found in two of the four specimens. In the 2007 specimen three clone sequences were 89% identical to an uncultured bacterium from a cold seep microbial mat, and 83% identical to *Spiroplasma eriocheiris* [Genbank accession number (acc. nr.): KU878561]. Also in this specimen one clone was found with 84% sequence identity to an uncultured bacterium or chloroplast from coral tissue (acc. nr: KU878562). In the 2008 specimen two clones were found with 99% sequence identity to *Thermus scotoductus* (acc. nr: KU878563).

**Figure 3:**
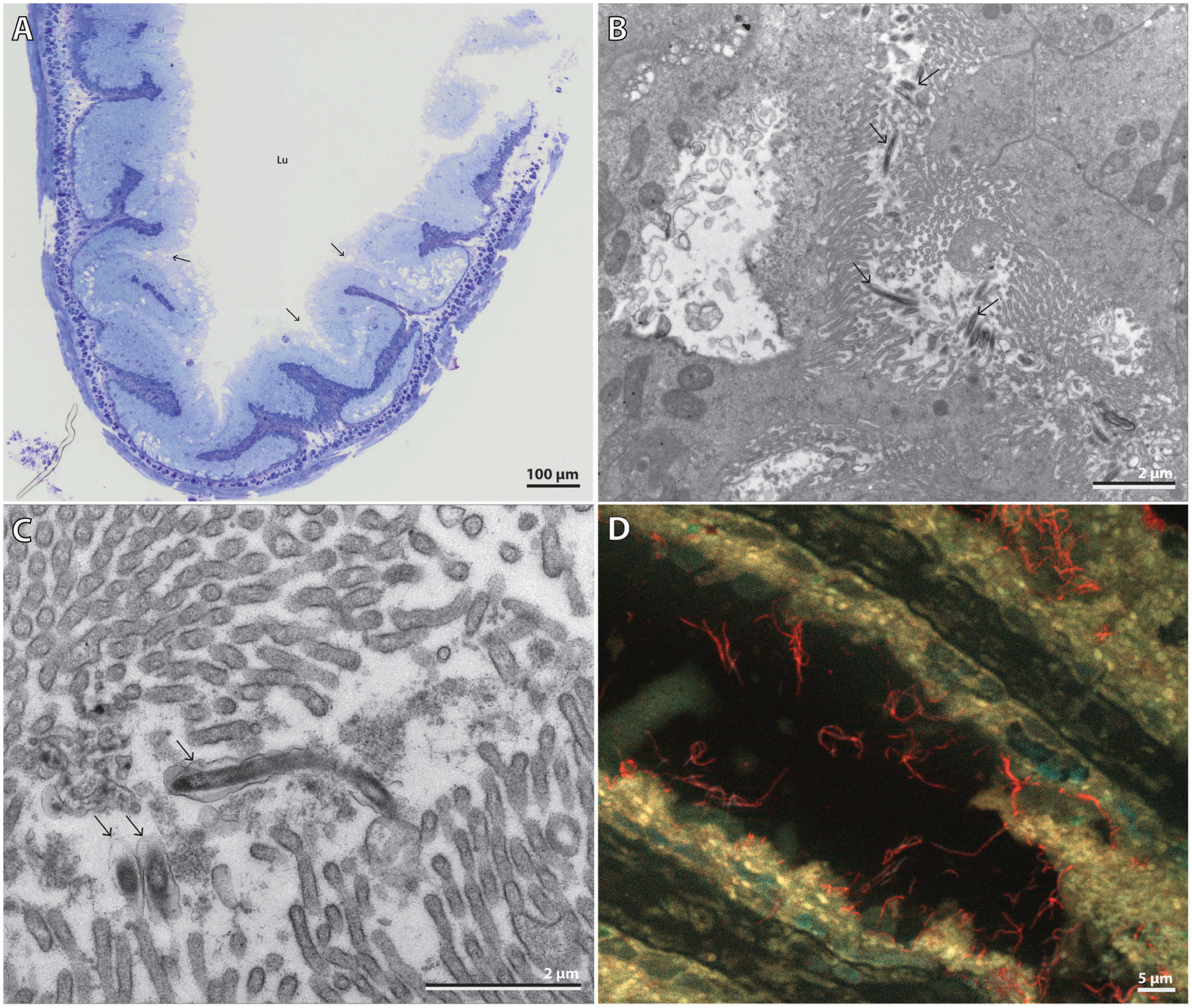
**(A)** Composite light microscopy image of half of a gut cross-section. Arrows point to the “pocket structures” where the symbionts were observed. Lu = gut lumen. Scale bar, 100 μm. **(B)** TEM micrograph of partially-visible elongated bacterial cells among host microvilli within pockets formed by the gut epithelium. Arrows point to various bacteria. Scale bar, 2 μm. **(C)** TEM micrograph close-up of three bacteria, one almost fully visible. Arrows point to the bacteria. Scale bar, 2 μm. **(D)** Extended focus multi-channel epifluorescence micrograph of a gut section hybridized with the symbiont-specific probe PricSym652-CY3 and stained with DAPI. *Cand*. T. priapulorum symbionts appear red, host nuclei cyan, and autofluorescent host tissue yellow. Scale bar, 5 μm.

Light microscopy and TEM of *P. caudatus* midgut sections showed a straight gut consisting of uniform epithelial cells with a microvillous border apically. Cilia and cuticular structures were absent. The gut epithelium was annulated and folded, forming “pockets” where the microvilli from adjacent folds came into contact (Figure 3A). TEM revealed uniform bacterial cells located among microvilli within such folds, and therefore only partially visible (Figures 3B,C), in agreement with the previous observation by McLean (1985). Their morphology was similar to that of the microvilli, i.e., elongated slightly curved rods, 0.2 × ≥3.5 μm in size, and surrounded by an outer folded membrane-like structure, although this may be a sample preparation artifact (Figure 3C). The ultrastructure of the host gut cells did not show any signs of necrosis; likewise, the bacterial cells appeared fully intact, suggesting that they were unaffected by the strongly proteolytic digestive fluids of the host (Nilsson and Fänge, 1967).

FISH with the specific probe PricSym652 identified these bacteria as being cells of the *P. caudatus*-specific phylotype and revealed, by extended-focus imaging, that many cells were ≥5 μm long (Figure 3D). Their affiliation to Alphaproteobacteria was confirmed with probe ALF1b (data not shown); few, if any, other bacterial cells were detected by FISH with the general bacterial probe or by DAPI staining (Figure S4), indicating that almost all other gut bacteria had been removed along with the gut content in the washing steps during gut preparation.

We acknowledge that only a small number of *P. caudatus* specimens has been analyzed due to the difficulty in sampling more animals. However, they were collected in different years and from distinct sites, and all of them contained the *P. caudatus*-specific phylotype. These putative symbionts showed a physical association to the gut epithelium strong enough to resist removal along with the general microbiota, caused no apparent damage to host tissue, and were not digested by the host; occasional phagocytosis may still be possible, as McLean (1985) observed yet unidentified bacteria in vacuoles in the intestinal cells. These collective findings indicate a stable, specific symbiosis of beneficial or commensal nature between the novel phylotype and *P. caudatus*.

Based on <90% 16S rRNA gene sequence identity to their closest relatives (Table 1), the symbionts represent a new species within a new genus (Yarza et al., 2014). Together with their specific identification by FISH with probe PricSym652, morphological description as elongate, curved rods of 0.2 × 5 μm size, their specific habitat in the gut of *P. caudatus*, and in accordance with the recommendations of Stackebrandt et al. (2002), we propose a new candidate genus and species for these as yet uncultured symbionts, with the name “*Candidatus* Tenuibacter priapulorum” gen. nov., sp. nov. [Te'nu.i.bac'ter. pri.a.pu.lo'rum. L. adj. *tenuis*, slender; Gr. n. *bacter*, rod; L. *priapulorum*, gen. pl. of *Priapulus*: slender rod-shaped bacterium belonging to the genus *Priapulus*].

**Table 1:** **Distance matrix of 16S rRNA gene identities (in %) for all members of the candidate family Tenuibacteraceae (1–13) and the type species of Holosporaceae (14) and Rickettsiaceae (15)**.

	**1**	**2**	**3**	**4**	**5**	**6**	**7**	**8**	**9**	**10**	**11**	**12**	**13**	**14**
1	100													
2	99.8	100												
3	99.8	99.7	100											
4	99.5	99.3	99.3	100										
5	89.6	89.5	89.7	88.9	100									
6	88.0	87.8	87.9	87.3	88.5	100								
7	88.6	88.4	88.5	87.3	88.3	87.4	100							
8	88.8	88.7	88.9	88.2	88.5	87.7	95.1	100						
9	85.5	85.4	85.5	85.0	85.7	84.6	85.4	85.7	100					
10	85.1	85.1	85.2	84.1	85.3	83.8	84.5	84.7	85.4	100				
11	85.3	85.1	85.1	83.8	85.0	83.3	84.7	84.8	85.3	93.3	100			
12	84.6	84.6	84.6	84.4	84.1	81.5	84.1	84.3	85.3	84.4	84.5	100		
13	82.4	82.3	82.4	82.4	83.5	80.9	82.9	82.7	84.8	84.6	84.9	84.0	100	
14	81.8	81.6	81.7	82.1	80.5	80.6	80.2	81.3	82.7	81.7	81.5	82.0	81.6	100
15	80.6	80.6	80.7	79.4	82.2	79.8	80.8	83.5	81.9	81.9	81.6	81.9	81.8	83.4

*Dark gray shading, Cand. Tenuibacter priapulorum; light gray shading, other members of Tenuibacteraceae; no shading, outgroup. 1, Cand. T. priapulorum 2011; 2, Cand. T. priapulorum 2007; 3, Cand. T. priapulorum 2008; 4, Cand. T. priapulorum 2004; 5, D. magna uncultured bacterium; 6, Cand. Hepatincola porcellionum; 7, E. imperialis uncultured gut bacterium; 8, D. opuntiae uncultured bacterium; 9, S. maritima uncultured bacterium; 10, E. sinensis uncultured gut bacterium DQ856522; 11, N. frasassianus uncultured bacterium; 12, T. putrecentiae uncultured bacterium; 13, E. sinensis uncultured gut bacterium DQ856561; 14, Holospora obtusa; 15, Rickettsia prowazekii*.

### A novel clade of putative gut symbionts

*Cand*. Tenuibacter priapulorum clusters within a well-defined but previously unrecognized clade of the order Rickettsiales (Alphaproteobacteria; Figure 2); with 16S rRNA sequence identities of <83% to its sister clades (Table 1), this newly defined clade represents a novel family (Yarza et al., 2014), for which we propose the candidate name Tenuibacteraceae.

This candidate family hitherto exclusively consisted of arthropod-associated-bacterial taxa. Except for the mitten crab *Eriocheir sinensis*, that contained two different sequence types, each arthropod host species appeared to harbor only one specific member of the clade. However, the crabs had been reared in aquaculture and fed other aquatic animals and innards (Li et al., 2007); thus the feed rather than the crab might be the actual source of the sequences, and the associations within the clade may still be species-specific symbioses. Host phylogenies did not correspond to the phylogenies of the respective putative symbionts (Figure 2 and Figure S1), suggesting that host and symbiont have not co-speciated. Vertical symbiont transfer and acquisition from arthropod prey may be possible, and may even have been the route of (an ancient) acquisition for the symbiont of the *P. caudatus*, given its terminal position in the symbiont clade (Figure 2), while the priapulid host is basal relative to arthropods (e.g., Borner et al., 2014 and Figure S1).

Many members of the Rickettsiales are intracellular symbionts (Fredricks, 2006), including Holosporaceae, the sister group of the newly defined clade, which consists of intranuclear symbionts of protists (Fredricks, 2006; Eschbach et al., 2009). The actual habitat or life style of the candidate family Tenuibacteraceae is less clear: sequences of five of its ten members were recovered from the digestive tract of arthropods and priapulids, while the remainder originated from unidentified tissues of whole arthropod specimens, including their gut (Table 2). The limited information available from *Cand*. Tenuibacter priapulorum, the woodlouse symbiont *Cand*. Hepatincola porcellionum (Wang et al., 2004), the orchid bee *Euglossa imperialis* (Koch et al., 2013) and the mitten crab (Li et al., 2007), however, suggests that members of candidate Tenuibacteraceae are extracellular digestive tract symbionts; their function remains elusive, also because of the vastly different habitats, lifestyles, and feeding modes of their hosts (Table 2). In general, gut symbioses of arthropods, best studied in insects (Engel and Moran, 2013), show large differences in symbiont diversity and function, precluding unifying predictions.

**Table 2:** **Taxonomy and ecology of host animals of the candidate family Tenuibacteraceae, and origin of their putative symbiont sequence**.

**Host (vernacular names)**	**Taxonomy**	**Habitat**	**Diet**	**Tenuibacteraceae sequence origin**
*Priapulus caudatus* (Penis worm)	Priapulida	Marine (benthic)	Generalist predator and scavenger	Midgut
*Porcellio scaber* (Rough woodlouse)	Arthropoda, Crustacea, Oniscidea	Terrestrial	Detritus, mainly plant litter	Hepatopancreas (digestive tract)
*Daphnia magna* (Water flea)	Arthropoda, Crustacea, Cladocera	Freshwater/brackish	Phytoplankton, filter-feeder	Unknown
*Euglossa imperialis* (Orchid bee)	Arthropoda, Insecta, Euglossini	Terrestrial	Nectar and pollen	Whole gut
*Dactylopius opuntiae* (Cochineal)	Arthropoda, Insecta, Coccoidea	Terrestrial	Plant sap	Entire specimen
*Eriocheir sinensis* (Chinese mitten crab)	Arthropoda, Crustacea, Brachyura	Freshwater/brackish (benthic)	Omnivore^a^	Whole gut
*Niphargus frasassianus* (Amphipod)	Arthropoda, Crustacea, Amphipoda	Freshwater/in caves (troglobitic)	Likely omni- and detritivore	Appendages^b^
*Strigamia maritima* (Centipede)	Arthropoda, Myriapoda, Chilopoda	Terrestrial/marine (littoral)	Predator of marine invertebrates	Whole genome sequencing of host
*Tyrophagus putrescentiae*^c^ (Mold mite)	Arthropoda, Arachnida, Acari	Terrestrial	Primarily fungivorous, pest of stored goods	Entire female specimen

a *Tenuibacteraceae sequence were only found in aquaculture crabs fed innards and aquatic invertebrates (Li et al., 2007); these may have been the actual symbiont source, which would explain the recovery of two distinct symbiont sequences from the mitten crabs*.

b *During a study targeting sulfur-oxidizing epibionts in the appendages (Bauermeister et al., 2012), a single Tenuibacteraceae sequence from a single specimen was obtained, which may have originated from contamination with fecal material during dissection*.

c *Putative symbiont sequence misclassified in the original publication (Qu et al., 2014) as Tistrella sp*.

With the addition of *P. caudatus*, the host range of the symbiont clade is expanded from Arthropoda to Ecdysozoa. It is important to note that the members of Ecdysozoa have microvilli-lined guts rather than cilia-lined as is the norm among other protostome animals (Giribet et al., 2000; Pechenik, 2015). Given the very different ecologies of the various hosts (Table 2), microvilli-lined guts may be their only common feature. Together with the similar elongate morphology and tissue affinity of *Cand*. Tenuibacter priapulorum and *Cand*. Hepatincola porcellionum (no data are available for the other symbionts), this observation suggests that the symbionts require microvilli to adhere and interact with their hosts and their host-range is limited to Ecdysozoa.

## Author contributions

PF, AS, and KK conceived the study and designed the work; PK, KK, PF, JN, and AS performed experimental work; PK, KK, and AS analyzed molecular data; all authors discussed and interpreted the results; PK, PF, and AS wrote the paper with editorial help of KK and JN.

## Funding

This study was funded by grants of the EU FP7 ASSEMBLE programme and the Danish Council for Independent Research Natural Sciences to AS, and by the Danish National Research Foundation. The Centre for Stochastic Geometry and Advanced Bioimaging is supported by the Villum Foundation.

### Conflict of interest statement

The authors declare that the research was conducted in the absence of any commercial or financial relationships that could be construed as a potential conflict of interest.
