# Supplementary material for: A Novel Extracellular Gut Symbiont in the Marine Worm Priapulus caudatus (Priapulida) Reveals an Alphaproteobacterial Symbiont Clade of the Ecdysozoa
Source: Front Microbiol. 2016 Apr 26;7:539. doi: 10.3389/fmicb.2016.00539 (PMC4844607; doi:10.3389/fmicb.2016.00539)
Supplement: Supplementary file 1 [file Image1.PDF]

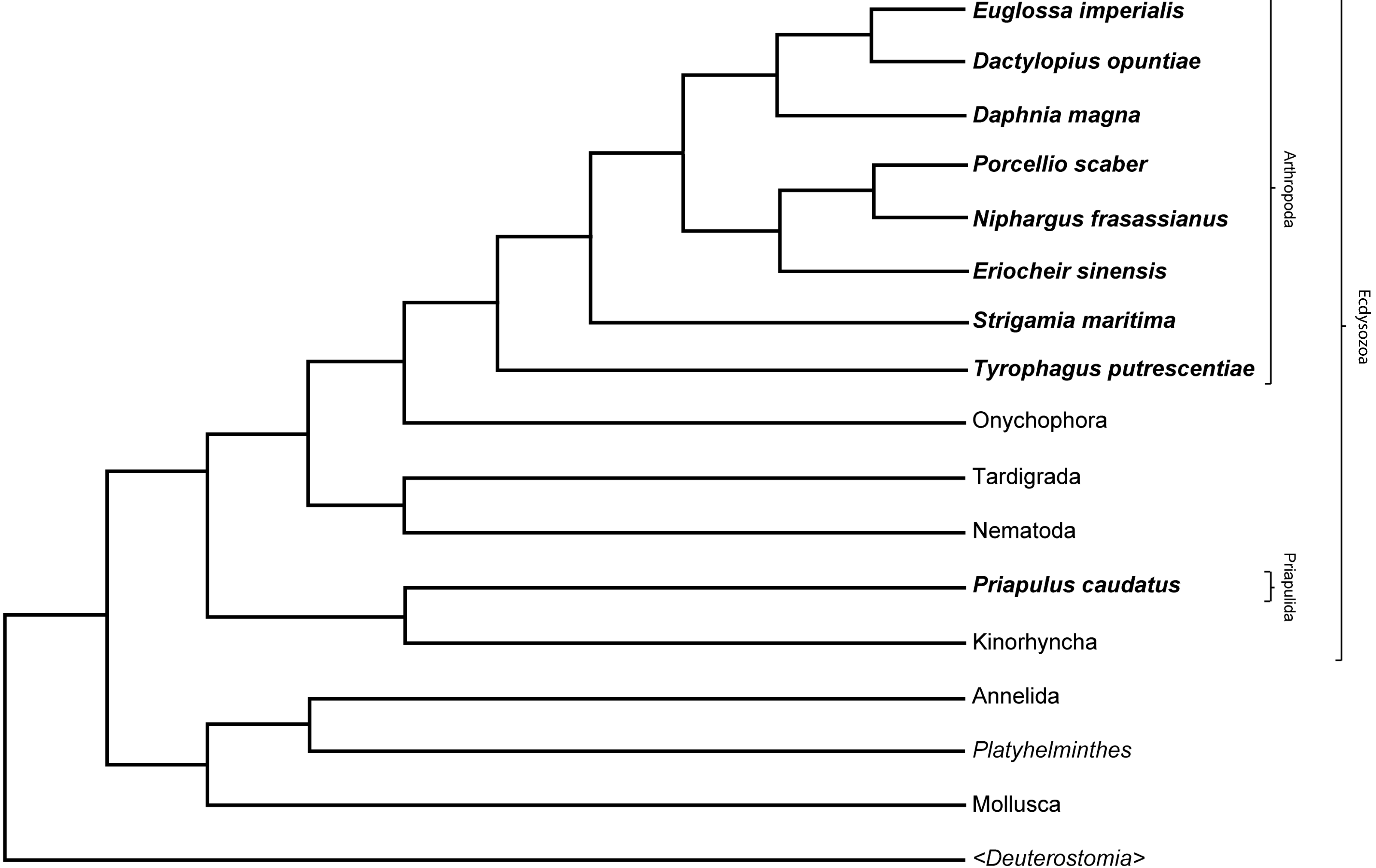

SUPPLEMENTARY FIGURE S1: Phylogeny of the host species for the candidate family Tenuibacteraceae among selected animal phyla. Cladogram modified from Borner *et al.* 2014.

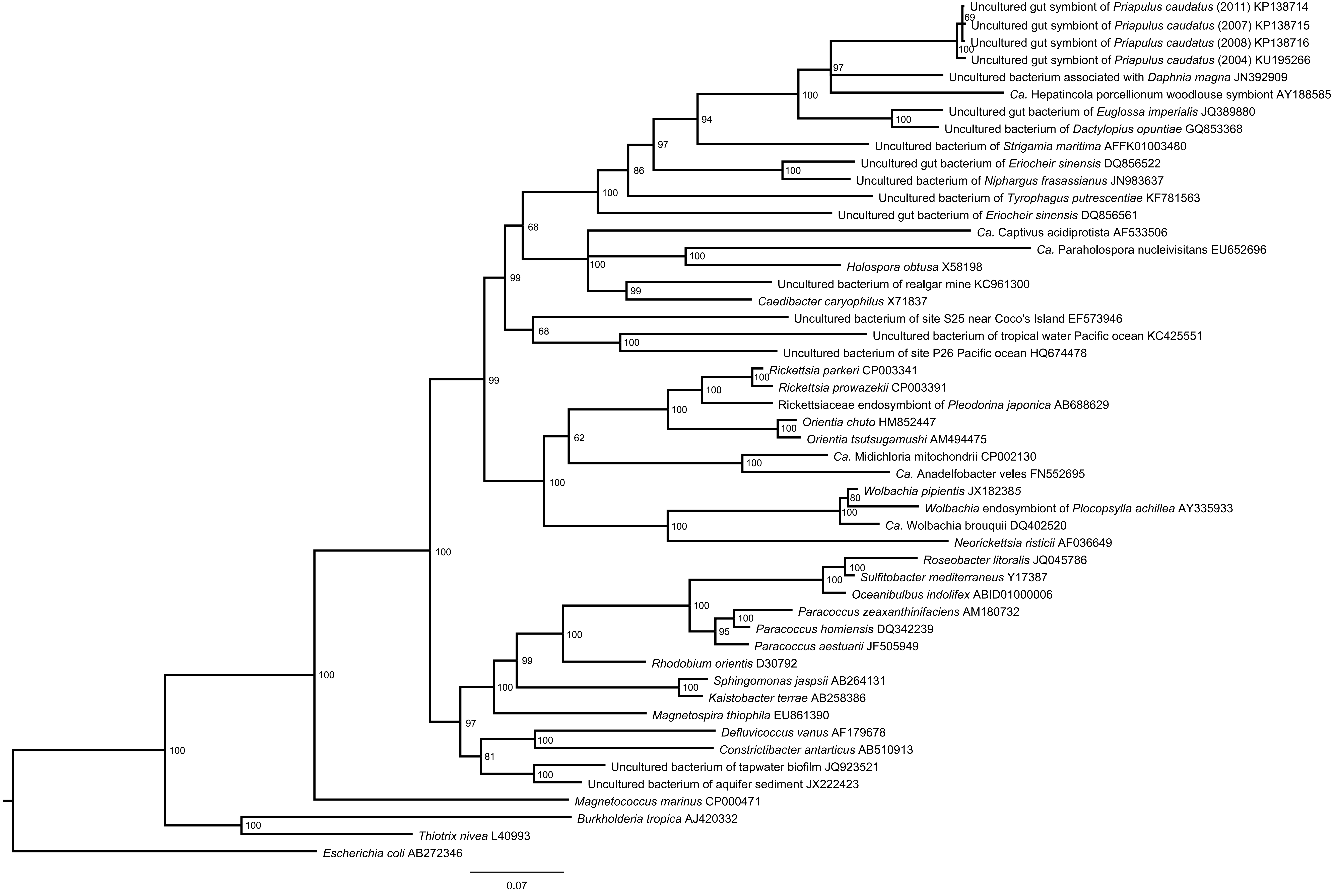

SUPPLEMENTARY FIGURE S2: BI phylogeny of the P. *caudatus* symbionts within the proposed candidate family Tenuibacteraceae and relative to other Alphaproteobacteria based on 16S rRNA gene sequence data. Node values represent posterior probabilities. Scale bar, 7% estimated sequence divergence.

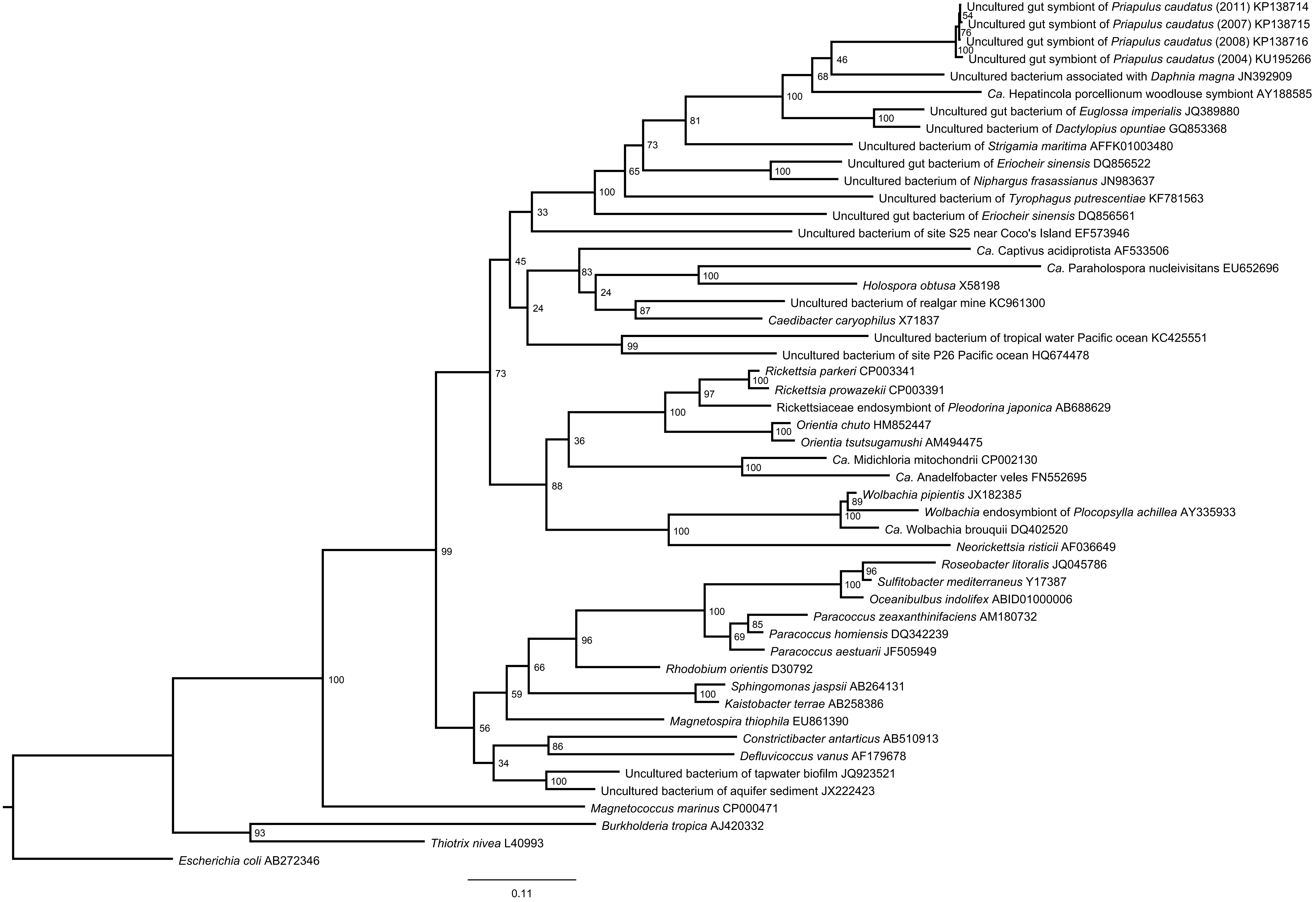

SUPPLEMENTARY FIGURE S3: ML phylogeny of the *P. caudatus* symbionts within the proposed candidate family Tenuibacteraceae and relative to other Alphaproteobacteria based on 16S rRNA gene sequence data. Bootstrap values (5000 iterations) are displayed at the nodes. Scale bar, 11% estimated sequence divergence.

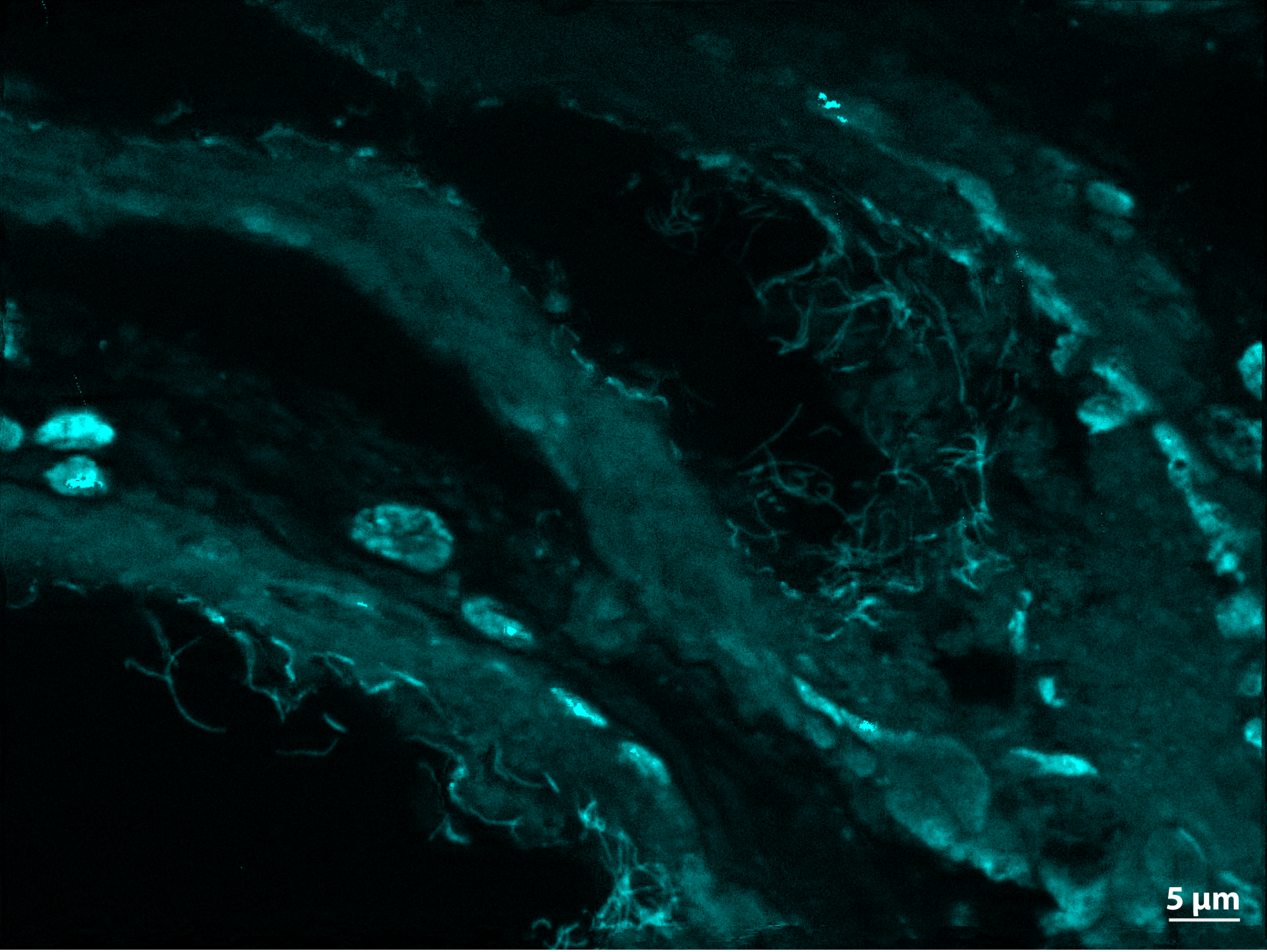

SUPPLEMENTARY FIGURE S4: Extended focus epifluorescence micrograph of a *P. caudatus* midgut section after DAPI staining. Host nuclei and *Cand.* T. priapulorum symbionts appear in light blue against a dark blue background. Scale bar, 5 µm.
